# Supplementary figures and images for: The distinct localization of CDC42 isoforms is responsible for their specific functions during migration
Source: J Cell Biol. 2024 Feb 22;223(3):e202004092. doi: 10.1083/jcb.202004092 (PMC10883850; doi:10.1083/jcb.202004092)

Fig 2B in manuscript:

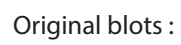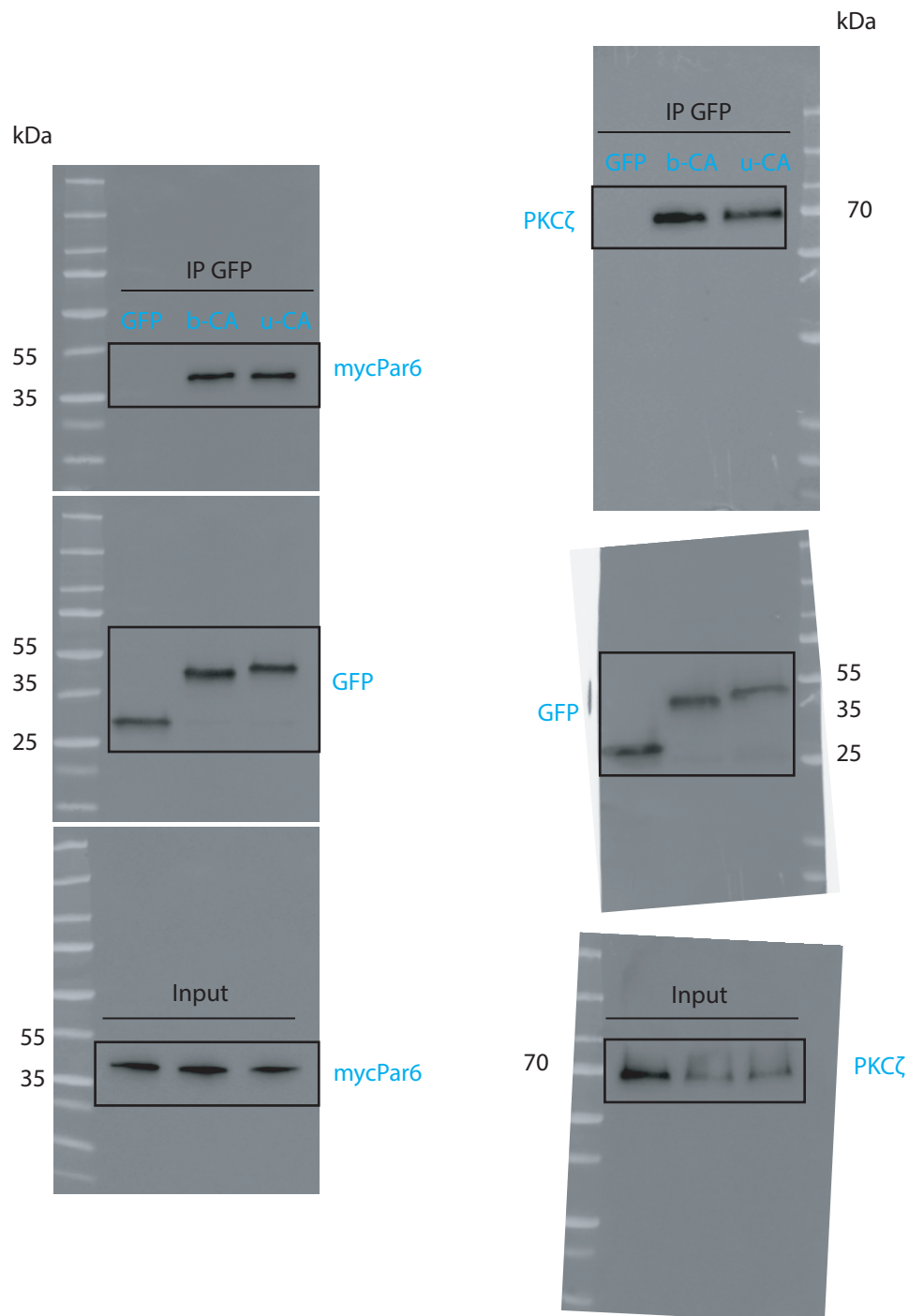

Supplement: SourceData F2 — is the source file for Fig. 2. [file JCB_202004092_SourceDataF2.pdf]

Figure 4C

Fig 4C in manuscript:

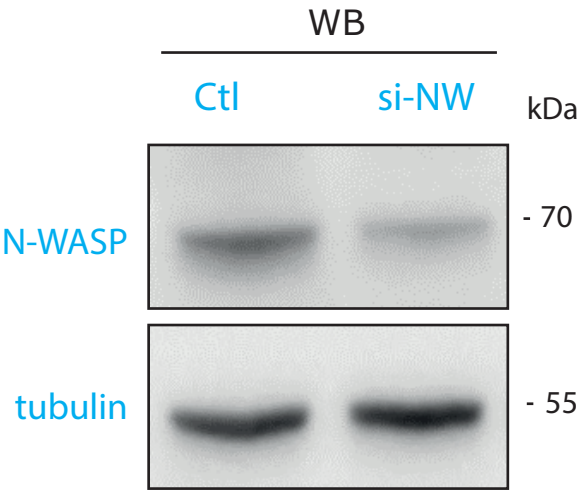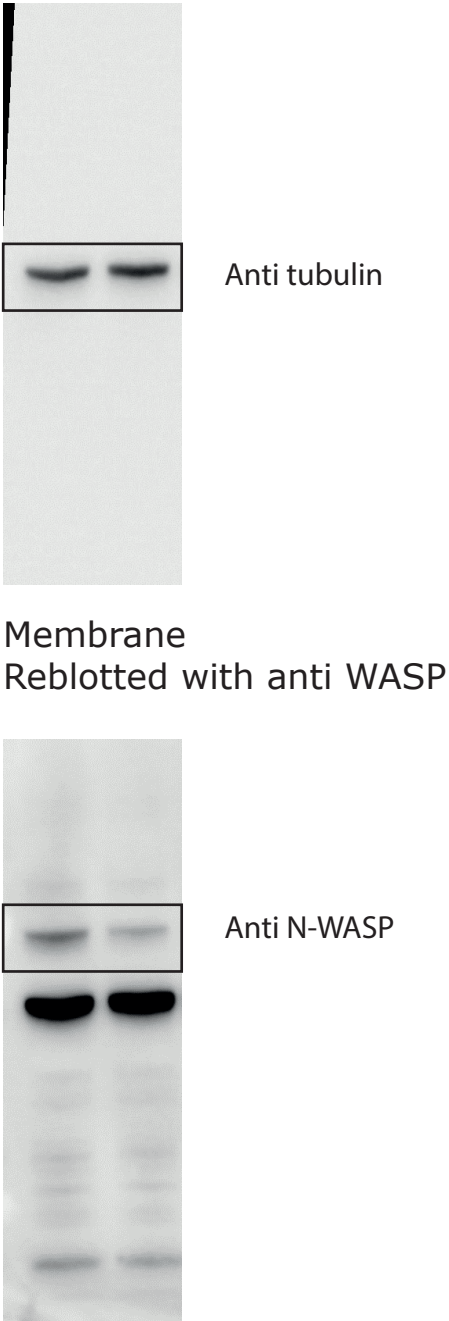

Figure 4G

Fig 4G in manuscript:

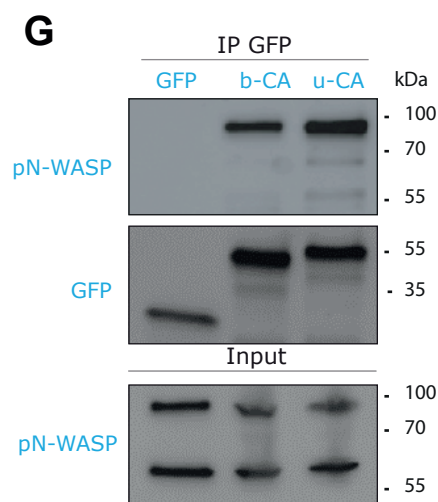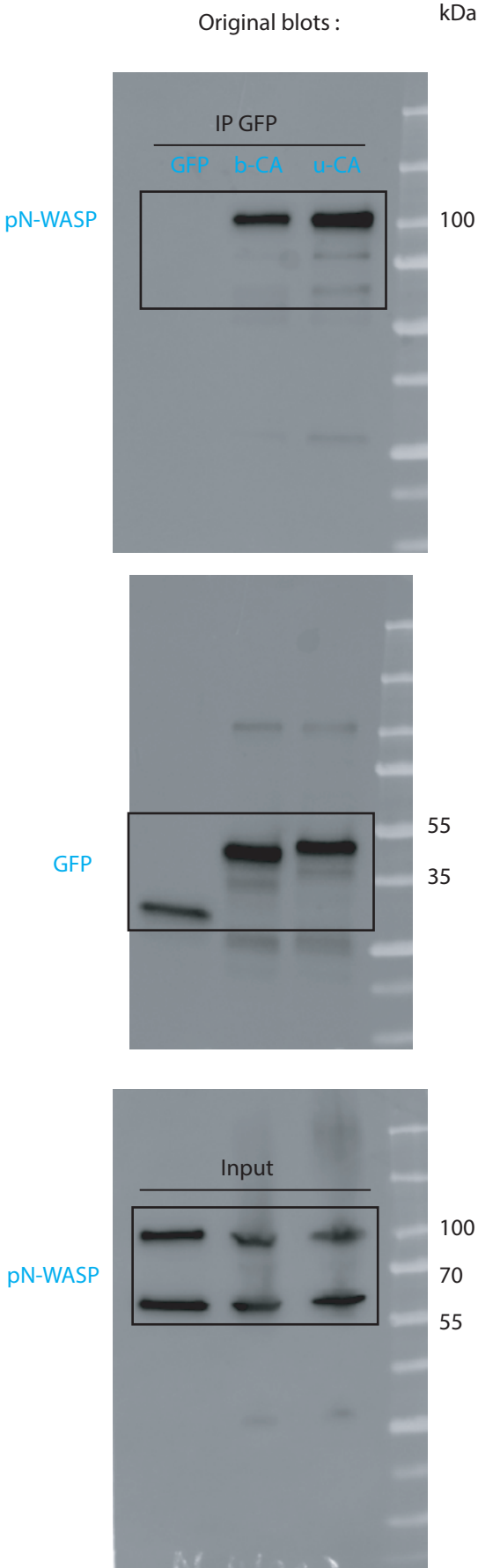

Supplement: SourceData F4 — is the source file for Fig. 4. [file JCB_202004092_SourceDataF4.pdf]
